# Vasorin is a potential serum biomarker and drug target of hepatocarcinoma screened by subtractive-EMSA-SELEX to clinic patient serum

**DOI:** 10.18632/oncotarget.3541

**Published:** 2015-03-12

**Authors:** Shaohua Li, Hui Li, Xiqin Yang, Wei Wang, Aixue Huang, Jie Li, Xingliang Qin, Fei Li, Guanyi Lu, Hongmei Ding, Xueting Su, Lvbin Hou, Wei Xia, Ming Shi, Hongwen Zhang, Qiang Zhao, Jie Dong, Xingfeng Ge, Leqiao Sun, Chenjun Bai, Chaonan Wang, Xuelian Shen, Tao Fang, Fusheng Wang, Heqiu Zhang, Ningsheng Shao

**Affiliations:** ^1^ Beijing Institute of Basic Medical Sciences, Beijing, China; ^2^ Beijing Institute of Pharmacology and Toxicology, Beijing, China; ^3^ Department of Interventional Radiology, General Hospital of Fuzhou, Fuzhou, China; ^4^ Center of Infectous Disease, Beijing 302 Hospital, Beijing, China

**Keywords:** vasorin, biomarker, hepatocarcinoma, subtractive-EMSA-SELEX, serum

## Abstract

We report a new biomarker of hepatocarcinoma, vasorin (VASN), screened by a subtractive EMSA-SELEX strategy from AFP negative serum of hepatocellular carcinoma (HCC) patients with extrahepatic metastases. VASN was verified to be highly expressed in sera of 100 cases of HCC patients compared with 97 cases of normal persons and 129 cases of hepatitis patients. Further validation by Q-PCR, IFA and Western blot showed higher expression of VASN at mRNA and protein levels in HCC cell lines and HCC tissues than in normal controls. RNA interference and forced overexpression assays verified that VASN promotes cell proliferation and migration and inhibits apoptosis. Down-regulation of microRNA miR145 and miR146a is an important mechanism leading to high expression of VASN. Conclusion: As a membrane protein and/or as free protein, VASN may be an effective target for biological treatment of liver cancer and is a potential biomarker for HCC diagnosis. Small molecular nucleotides targeting VASN are promising biological therapies to HCC.

## INTRODUCTION

Alpha-Fetoprotein (AFP) is by far the most commonly used serological biomarker in clinical practice for liver cancer screening, early diagnosis, evaluation of therapeutic efficacy and prognosis [1, 2]. However, the clinical diagnostic accuracy of AFP is unsatisfactory due to low sensitivity and specificity. About 30% to 40% of patients with hepatocellular carcinoma (HCC) are serum AFP negative clinically, a growing trend in recent years [3]. These patients get poor diagnosis, prognosis and efficacy evaluation for lack of effective liver cancer tumor markers. Therefore, there is an urgent need for developing new HCC biomarkers either independent of or combined with AFP for diagnostic, prognostic or predictive value [4, 5].

Many biotechnology methods such as genomics, RNomics, proteomics, metabolomics and antibody chip technology have been widely used for screening and identification of new tumor biomarkers [6-9]. Unfortunately, until now, relatively few tumor markers of clinical diagnostic value have been available due to poor specificity and unsatisfactory reproducibility. Methodological limitations may be the result of protein enzymolysis or loss during sample preparation and the unsuitability of some special samples containing low abundance protein or strongly alkaline proteins. By providing specific recognition, monoclonal antibody chip-based assays can be used for the isolation of cancer biomarkers, but they are limited by difficulties in generating tumor-specific antibodies. Aptamer-Facilitated Biomarker Discovery (AptaBiD) is a promising technology for biomarker discovery [10-12]. AptaBiD is based on individual or pooled aptamers, a new class of molecular probe for specific recognition of different molecular targets. Aptamers (from the Latin aptus - fit, and Greek meros - part) are short single-stranded DNA (ssDNA) or RNA molecules with high affinity to a wide variety of targets, ranging from small organic molecules to complex proteins. Aptamers' affinity is attributed to their specific three-dimensional shapes. They are evolved through an *in vitro* selection process known as systematic evolution of ligands by exponential enrichment(SELEX), a high-flux screening technique that involves the progressive selection of highly specific ligands by repeated rounds of selection and amplification from a large random synthetic nucleic acid library [13, 14]. Because of their discriminate recognition which rivals that of commonly used antibodies [15], easy synthesis and modification, less severe immunogenicity and small size, aptamers are of growing interest in biotechnological and therapeutic applications. AptaBiD was first developed to isolate cell biomarkers and we previously developed it for tissue biomarkers [16], but here we utilize AptaBid to identify a clincally non-invasive serum biomarker.

AptaBiD involves three major stages: (i) differential selection of aptamers or pools of aptamers through SELEX; (ii) aptamer-based isolation of biomarkers; and (iii) mass spectrometry identification of biomarkers. Usually, the first stage involves a counter selection that is guaranteed to generate aptamers that differentiate selection targets from counter-selected partners which are highly homologous to targets but show some distinctive molecular differences [17-19]. The important feature of the counter selection is that it does not require prior knowledge of the complex and is not restricted by the properties of the targets, providing a new approach for biomarker verification by the obtained specific aptamers. Another crucial step for a successful selection is the separation of target bound oligonucleotides from unbound oligonucleotides. Unlike selection to intact biological entities such as cells, organelles and tissues, for which bound oligonucleotides can be easily separated from free ones by centrifugation or through binding to mounted tissue slides, the separation is a more challenging task for soluble complex targets such as serum. Most methods use target immobilization on nitrocellulose, magnetic beads or column materials, e.g. sepharose or agarose [20]. However, either substantial amounts of target molecules are necessary to achieve sufficiently high loading of the column, or pre-removal of highly abundant non-target proteins are required because of limited binding capacities of medium such as nitrocellulose and magnetic beads, especially for selection of trace level targets in a very complex system such as cell lysate and serum. Sahar Javaherian et al report a new separation with non-equilibrium capillary electrophoresis of equilibrium mixtures [21, 22], but this method needs a capillary electrophoresis (CE) apparatus equipped with a fluorescence detector and requires sufficiently high target levels in lysate. Here, we report using subtractive-EMSA which separates bound nucleotides with free ones according to gel mobility rates. It requires only very small amounts of target, no pre-treatment of targets or specific apparatus. For the positive selection, we recover DNA from gel that shifts more slowly than the free library; for the negative selection, we recover DNA identical to the free library. We developed a subtractive liquid SELEX, subtractive-EMSA-SELEX, targeted AFP negative serum sample of HCC patients with extrahepatic metastases for HCC serum biomarker identification. Using normal serum as a counter target in the selection round and using EMSA to separate aptamers bound with serum differential molecules from free nucleotides, enriched pools were obtained and verified to discriminate HCC serum and normal serum after 5 rounds of selection. The enriched pool was analysed by MS to identify candidate biomarkers. In this report, we will focus on one of these, vasorin, for its biomarker validation and biological function.

Vasorin (VASN) was first elucidated as a cell surface and secreted protein that modulates the arterial response to injury through inhibiting the TGF-β signaling pathway [23, 24]. It is expressed at its highest levels in the aorta, at intermediate levels in kidney and placenta tissue and at its lowest levels in brain, heart, liver, lung and skeletal muscle tissue. VASN is further identified as a substrate of ADAM17, a metalloprotease, which generates a soluble fragment encompassing the extracellular domain of VASN, which inhibits TGF-β signaling [25]. Despite the importance of TGF-β in tumor progression, the status and function of VASN in tumors are seldom reported. Caccia et al identified VASN as a predictive biomarker that is sensitive to RPI-1 and dasatinib treatments in thyroid cancer cells by analyzing tumor cell-line secretomes [26]. VASN is also reported as a HIF-1 target that regulates mitochondrial redox pathways and as a potential diagnostic marker and therapeutic target in human glioblastoma [27]. There is no report of vasorin in hepatocarcinoma until now. We report herein first that VASN is a prospective biomarker of HCC. We next find that down-regulated microRNA miR145 and miR146a are an important mechanism leading to high expression of VASN. Finally, small nucleotide (siRNA or aptamers) targeting VASN could be promising therapeutics for HCC.

## RESULTS

### Identification of VASN as a differentially expressed protein from AFP negative and positive serum samples from HCC patients by subtractive – EMSA - SELEX

**Figure 1:**
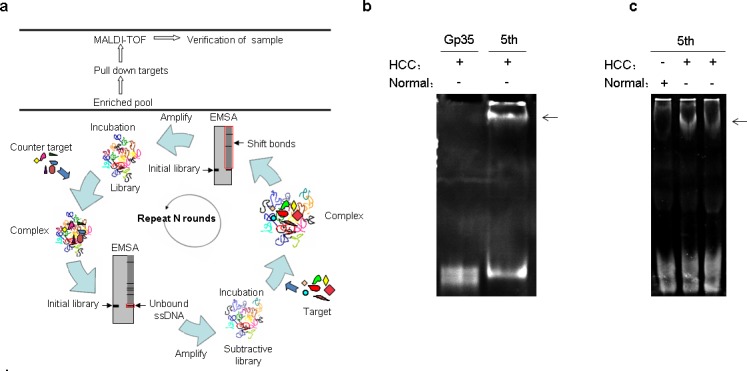
VASN is identified as a candidate biomarker from AFP negative serum samples of HCC patients by subtractive-EMSA-SELEX (a) Schematic showing selection of specific aptamers by subtractive-EMSA-SELEX from liquid serum samples. The red box indicates in-gel ssDNAs which should be collected and amplified for the next round selection. (b) The fifth pool demonstrated an enrichment and discrimination for the serum sample used for selection. (c) The fifth pool demonstrated an enrichment and discrimination for different serum samples. The arrows indicate the complex band of aptamer and targets.

To identify HCC serum biomarker, we developed a subtractive - EMSA-SELEX strategy targeting AFP negative serum samples of HCC patients with extra hepatic metastases (Fig. 1a). After each round of selection, the enriched pool was labeled with FAM (carboxyfluorescein) by a modified 5′ primer through unequal length PCR. Enrichment of each round of selection was monitored by EMSA of the labeled pool and the targets or the counter targets complex. Selection rounds were repeated until there was an obvious binding of enriched pool with targets and until the enriched pool could discriminate targets from counter-targets. In this study, the fifth pool demonstrated an obvious enrichment and discrimination between counter target and target as well as between normal serum and AFP negative HCC serum (Figs. 1b, 1c).

The slowly migrated band of enriched fifth pool and target serum complex in EMSA was excised and the protein components in it were recovered and subjected to mass spectrometry analysis. The corresponding position in the control lane (containing the initial library and target serum complex) was excised and identified by MS simultaneously to exclude proteins of similar mobility to the complex. We performed MALDI-TOF MS (Matrix-Assisted Laser Desorption/Ionization Time of Flight Mass Spectrometry) followed by secondary peptide sequencing MS analysis for the identification of the mixture proteins. Significant protein hits had individual ions scores greater than 38, indicating identity or extensive homology (p<0.05) (Supplementary Table 1, Table 1). Among these potential biomarkers, peptides identified in Chain A, Apo-Human Serum Transferrin (Non-Glycosylated) also match transferrin (gi|4557871) that is also in the control band. Ceruloplasmin, Complement factor B or complement component C3 had already been reported elevated in hepatocarcinoma and other cancers [28-30]. The protein VASN showed a relatively high score probability and has never been studied on HCC.

**Table 1:** Potential biomarkers identified by MS

GI	Protein	Matched Peptides	Peptide	Proteins (GI) matching the same set of peptides
gi|1620909	Ceruloplasmin	5	K.DIASGLIGPLIICK.K+ (C) K.VNKDDEEFIESNK.M K.DLYSGLIGPLIVCR.R+ (C) K.LISVDTEHSNIYLQNGPDR.I R.GPEEEHLGILGPVIWAEVGDTI R.V	ceruloplasmin precursor or isoforms: (gi|4557485) (gi|119599289) (gi|119599290) (gi|126031006) (gi|158255874) (gi|221042622)
gi|291922	complement factor B	4	K.VASYGVKPR.Y K.VSEADSSNADWVTK.Q R.DFHINLFQVLPWLK.E K.EAGIPEFYDYDVALIK.L	Complement factor B preproprotein or Segment (gi|13278732) (gi|58176651) (gi|67782358) (gi|134105218) (gi|194384366) (gi|239781743) (gi|251837060)
gi|194384410	unnamed protein product	3	K.EYVLPSFEVIVEPTEK.F K.VQLSNDFDEYIMAIEQTIK.S R.EPGQDLVVLPLSITTDFIPSFR.L	complement component C3 or precursor (gi|179665) (gi|115298678)
gi|15489339	VASN protein	1	R.LLLLDLSHNSLLALEPGILDTANVEALR.L	CSRV314 or slit-like 2 precursor (gi|37181716) (gi|37181718) (gi|88702793)
gi|106529	Ig kappa chain C region	1	K.SGTASVVCLLNNFYPR.E+ (C)	Immunoglobulin (gi|125145) (gi|184848) (gi|185925) (gi|185927) (gi|185947) (gi|185949) (gi|229526) etc
gi|386789	hemopexin precursor	3	R.RLWWLDLK.S R.YYCFQGNQFLR.F+ (C) K.EVGTPHGIILDSVDAAFICPGSSR.L+ (C)	hemopexin precursor or unnamed protein (gi|1335098) (gi|11321561) (gi|189053897)
gi|51173528	carboxypeptidase N precursor	1	K.TLNLAQNLLAQLPEELFHPLTSLQTLK.L	Carboxypeptidase N, polypeptide 2 (gi|52788240) (gi|119598461) (gi|145207281) (gi|256217721)

### VASN has verified high expression in HCC patient sera, tissues and cell lines

**Figure 2:**
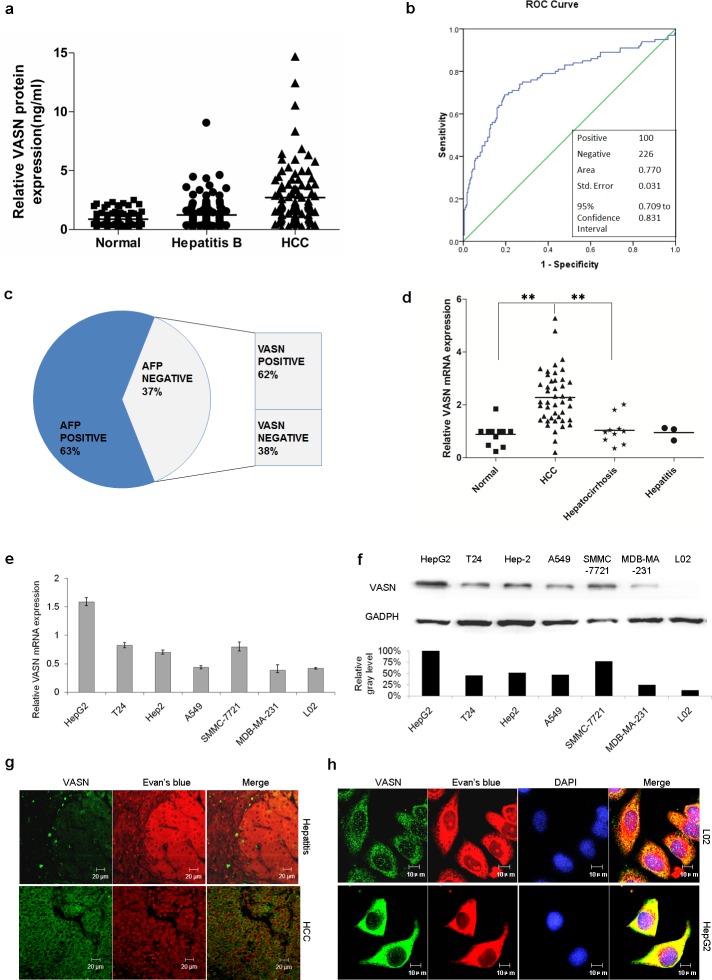
VASN is highly expressed in HCC patient serum, tissues and cell lines (a) VASN was verified to be high in HCC sera by a quantitative ELISA assay. The results confirmed the elevation of circulating VASN of HCC patients compared to that of control cohorts. (b) The ROC curve was generated and the area under the curve (AUC) is 0.770. (c) Among the 37 cases of AFP negative serum (37%), 62% of samples were VASN positive (≥ 1.5061ng/ml). (d) VASN mRNA was more highly expressed in hepatocarcinoma tissues than those in hepatocirrhosis, hepatitis and pericarcinoma tissues. (e) VASN mRNA was highly expressed in human hepatoma HepG2, SMMC-7721 cells as measured by real-time PCR. (f) The protein level of VASN was relatively high in human hepatoma HepG2, SMMC-7721 cells measured by Western blot. (g) The expression and localization of VASN protein were verified on HCC tissue slides and benign lesion (hepatitis) liver tissue slides by an indirect immunofluorescence assay. (h) VASN was located on the hepatocellular carcinoma cell surface according to immunofluorescence staining.

We collected sera from 100 proven cases of HCC, 129 cases of hepatitis B and from 97 normal individuals. The VASN level was determined by quantitative ELISA as described in the methods section. The results confirmed the elevation of circulating VASN of HCC patients compared to that of the control cohorts, (Fig. 2a, Supplementary Table 2). The area under the curve (AUC) of the receiver operating characteristic (ROC) curve by SPSS17.0 software was 0.770 (Fig. 2b). The cut-off value was set up as 1.5061ng/ml based on the Youden index so that the sensitivity reached 69% with a specificity of 80.5%. Furthermore, among the 37 cases of AFP negative sera (37%), 23 cases (62%) were VASN positive (≥ 1.5061ng/ml) (Fig. 2c), which suggested that combining the use of AFP and VASN could improve the sensitivity of liver cancer diagnosis. In this report, the diagnostic sensitivity would be increased to 86% from 63% and 69% if combined use of AFP and VASN respectively compared to their use independently.

Tissue cDNA chips that were β-actin calibrated included 68 cases of liver cancer tissue, pericarcinoma tissues and hepatocirrhosis. The expression of VASN mRNA was measured by real-time PCR. VASN mRNA levels in one pericarcinoma tissue that was relatively highly expressed in the control group was used as an external reference. The results confirmed that VASN mRNA was more highly expressed in HCC tissues than those in hepatocirrhosis, hepatitis or pericarcinoma tissues (Fig. 2d), providing additional evidence for vasorin as a potential HCC marker.

The expression of VASN mRNA and protein in various human cancer and normal cell lines was measured by real-time PCR and Western blot respectively. The results confirmed that vasorin was relatively highly expressed in human hepatoma HepG2, SMMC-7721 cells (Figs. 2e, 2f).

The expression and localization of VASN protein were verified for 7 cases of HCC and benign lesion (virus hepatitis and hepatocirrhosis) liver tissue slides as well as coverslips with adherent hepatic cancerous and normal cells by an indirect immunofluorescence assay. Evans blue contrast staining was used to reduce the background of non-specific autofluorescence and to help distinguish the specific FITC labelling from the red background [17]. We quantitated the vasorin in situ expression by staining as: (−) = <5% of cells stained; (+) = 5%-25% of cells stained; (+ +) = 26%–50% of cells stained; (+ + +) = 51%–75% of cells stained; and (+ + + +) = > 75% of cells stained. The semi-quantitative results showed higher expression of VASN in HCC and lower expression in benign lesions (Supplementary Table 3). Expression was mainly located in the cytoplasm, cell surface and cell gaps (Fig. 2g). In situ cell detection showed a consistent result (Fig. 2h).

### VASN increases hepatoma cell proliferation and migration and inhibits apoptosis

**Figure 3:**
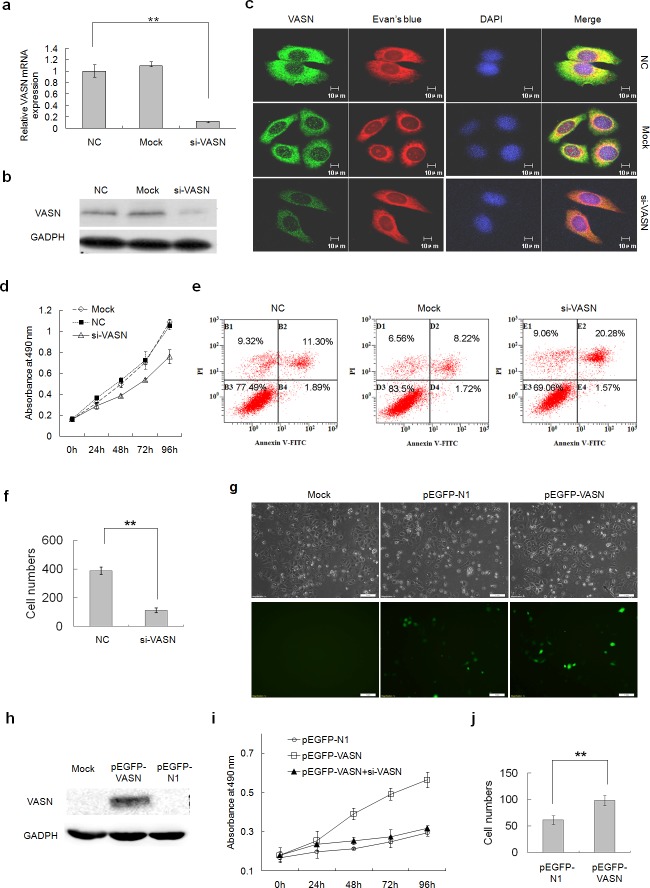
VASN increases hepatoma cell proliferation and migration and inhibited cell apoptosis (a) The knockdown effect of siRNA targeting VASN was confirmed at the mRNA level by q-PCR and (b) at the protein level by Western blot and (c) by immunofluorescence in HepG2 cells. (d) Knockdown of VASN in HepG2 cells resulted in decreased cell proliferation. (e) Knockdown of VASN in HepG2 cells resulted in increased apoptosis. (f) Knockdown of VASN in HepG2 cells resulted in reduced migration. (g) Over-expression of full-length GFP-tagged VASN in L02 cells was confirmed by fluorescence microscopy,and (h) Western blot. (i) Overexpression of full-length GFP-tagged VASN in normal L02 cells resulted in increased cell proliferation measured by the MTS assay, which VASN-specific siRNA can abolish. (j) Transwell chamber migration assay confirmed that overexpressed VASN allows increased cell migration in L02 cells.

The function of highly expressed VASN was explored by silencing VASN expression with siRNA in cancerous HepG2 cells and forced VASN overexpression in normal L02 cells. Proliferation was measured by MTS (3-(4,5-dimethylthiazol-2-yl)-5-(3-carboxymethoxyphenyl)-2-(4-sulfophenyl)-2H-tetrazolium) viability assays, apoptosis was measured by flow cytometry assays, and migration was measured by transwell chamber assays 48-96 hours after transfection. Silencing expression of VASN in HepG2 cells (Figs. 3a, 3b, 3c) resulted in decreased cell proliferation (Fig. 3d), increased apoptosis (Fig. 3e) and reduced migration (Fig. 3f) compared with the control groups. Forced overexpression of full-length GFP-tagged VASN in normal liver cells (Fig. 3g, 3h) resulted in increased cell proliferation which can be abolished by VASN specific siRNA (Fig. 3i). Transwell chamber migration assays confirmed that overexpressed VASN causes increased cell migration in normal liver cells (Fig. 3j). Since VASN is expressed on the cell surface, these results suggested that VASN could be an effective biological target of cancer treatment.

### Low levels of miR145 and miR146a are negatively correlated with the level of VASN in hepatoma cells and HCC patient sera

**Figure 4:**
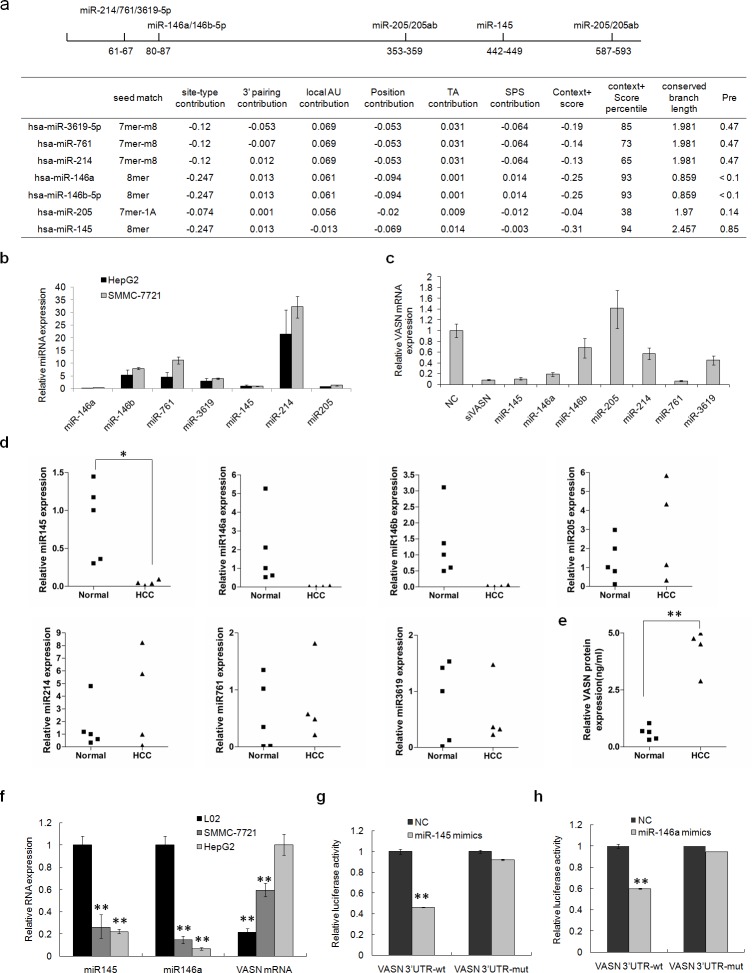
Low levels of miR145 and miR146a in hepatoma cells and HCC patient sera have a negative correlation with the level of VASN in those cells and sera (a) 7 miRNA potential target sites of the 3′ UTR of VASN were analyzed with TargetScan5.1 software. (b) The expression levels of 7 potential miRNAs in hepatoma cell lines HepG2 and SMMC-7721 were analyzed by Q-PCR. (c) The expression level of VASN mRNA was down regulated after transient transfection of miR145, miR146a and miR761 mimics into HepG2 cells. (d) The levels of 7 miRNAs in 5 cases of AFP negative HCC patient sera and 4 cases of normal serum samples by Q-PCR. (e) The VASN protein level was quantified in normal serum samples and HCC patient sera by ELISA. (f) The negative relationship of miR145 and miR146a to VASN mRNA levels was verified in hepatocellular carcinoma cell lines HepG2 and SMMC-7721 and normal liver cells L02 by Q-PCR. (g) miR145 could down regulate the luciferase activity of reporter vectors carrying wild type VASN 3′ UTR but had no effect on that of the miR145 mutant report vector. (h) miR146a down regulates the luciferase activity of reporter vectors carrying wild type VASN 3′ UTR but had no effect on that of the miR146a mutant reporter vector.

It is well known that miRNAs play important roles in regulating protein expression in cells. They even are mentioned as a new class of biomarker for tumor detection because of their abnormal expression in tumors. To identify which miRNAs might functionally target VASN in hepatoma cells, bioinformatic analysis with TargetScan5.1 was completed. There were 7 potential miRNA target sites in the 629bp full length 3′ UTR of VASN mRNA: miR146a, miR146b, miR145, miR205, miR214, miR761 and miR3619 (Fig. 4a). Among these miRNAs, real-time PCR indicated that miR146a, miR145, miR205 and miR3619 had relatively lower expression in hepatoma cell lines of HepG2 and SMMC-7721, which have high expression of VASN (Fig. 4b). VASN mRNA levels were significantly reduced after transient transfection of mimics of miR145, miR146a and miR761 into HepG2 cells (Fig. 4c). Furthermore, we examined the levels of the above 7 miRNAs in clinical HCC patient sera. We found that the levels of miR145, miR146a and miR146b were lower in 5 cases of AFP negative hepatoma patients sera compared with those in 4 cases of normal sera (Fig. 4d), showing a negative correlation with the expression level of VASN (Fig. 4e). This negative correlation was further verified in hepatocellular carcinoma cells HepG2, SMMC-7721 and normal liver cells L02 on the mRNA level (Fig. 4f). All these results suggested that VASN could be a functional target of miR145 and miR146a, and that low expression of miR145 and miR146a may result in the abnormally high expression of VASN in hepatoma cells.

A dual-luciferase reporter gene assay was performed to test our hypothesis. We constructed a series of PGL3 reporter gene vectors carrying wild type VASN 3′ UTR fragment or mutant fragments of miRNA target sites. The reporter vectors and miRNA mimics were co-transfected into HepG2 cells. The results showed that miR145 and miR146a could down regulate the luciferase activity of report vectors carrying wild type VASN 3′ UTR, but had no effect on the miR145 mutant VASN 3′ UTR or the miR146a mutant VASN 3′ UTR reporter vectors (Figs 4g, 4h).

### Overexpression of miR145 and miR146a downregulated cell growth and migration and increased cell apoptosis through downregulation of VASN expression in HepG2 cells

**Figure 5:**
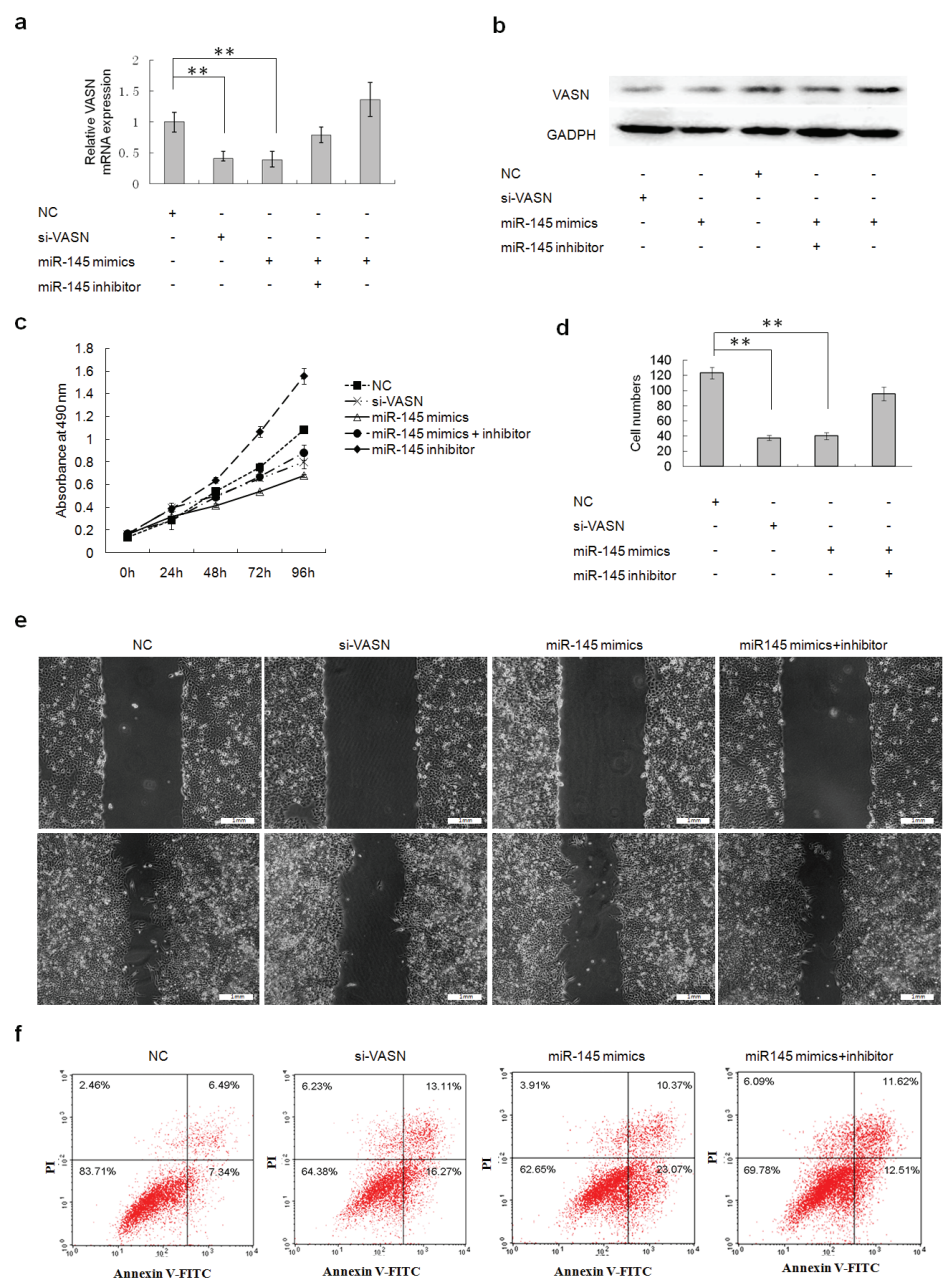
Transient overexpression of miR145 downregulated cell growth and migration and increased apoptosis through downregulation of VASN expression in HepG2 cells (a) The expression level of VASN mRNA was down regulated after transient transfection of miR145 mimics into HepG2 cells by real-time PCR. (b) The expression level of VASN protein was down regulated after transient transfection of miR145 mimics into HepG2 cells by western blot. (c) Overexpression of miR145 inhibited HepG2 cell growth by MTS assay. (d) Overexpression of miR145 mimics inhibited HepG2 cell migration by transwell chamber migration assay. (e) Overexpression of miR 145 mimics in HepG2 inhibited migration in a scratch wound assay. Upper: A scratch was created with a 200 ul-pipette tip and was photographed. Lower: Photographs at 36h after scratching. (f) Overexpression of miR145 induced HepG2 cell apoptosis.

**Figure 6:**
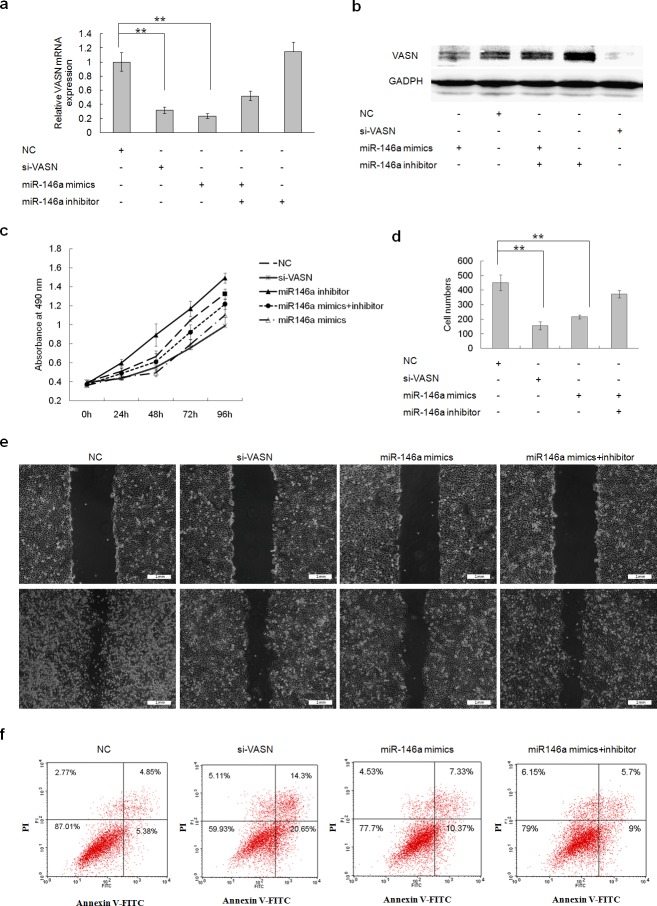
Forced Overexpression of miR146a downregulated cell growth and migration and increased cell apoptosis through downregulation of VASN expression in HepG2 cells (a) The expression level of VASN mRNA was down regulated after transient transfection of miR146a mimics into HepG2 cells by real-time PCR. (b) The expression level of VASN protein was down regulated after transient transfection of miR146a mimics into HepG2 cells by Western blot. (c) Overexpression of miR146a inhibited HepG2 cell growth by MTS assay. (d) Overexpression of miR146a inhibited HepG2 cell migration by transwell chamber migration assay. (e) Overexpression of miR 146a mimics in HepG2 inhibited migration in a scratch wound assay. Upper: A scratch was created with a 200 μl-pipette tip and photographed. Lower: Photographs at 36h after scratching. (f) Overexpression of miR146a induced HepG2 cell apoptosis.

The expression levels of VASN mRNA and protein were down regulated after transient transfection of miR145 and miR146a mimics into HepG2 cells (Figs. 5a, 5b, Figs. 6a, 6b). Cells underwent similar changes as observed in the VASN silencing experiment: cell proliferation was reduced (Fig. 5c, Fig. 6c), cell migration decreased (Figs. 5d, 5e, Figs. 6d, 1e) and apoptosis increased (Fig. 5f, Fig. 6f). Co-transfection of miRNA inhibitors could rescue those phenotypes to a certain degree (Fig. 5, Fig. 6). These results lay the foundation for the application of VASN-targeting nucleic acid drugs such as siRNA, miRNA, and aptamers in future treatment of high-VASN expressing hepatoma.

## DISCUSSION

AFP is by far the most commonly used serological biomarker in clinical practice for liver cancer screening, early diagnosis, evaluation of therapeutic efficacy and prognosis [18, 19]. However, the clinical diagnostic accuracy of AFP is unsatisfactory due to low sensitivity and specificity. About 30% to 40% of patients with HCC are serum AFP negative clinically, a growing trend in recent years [20]. These patients receive poorer diagnosis, prognosis and efficacy evaluation for lack of a more effective liver cancer tumor marker. Therefore, there is an urgent need to develop new HCC biomarkers either independently of or combined with AFP for diagnostic, prognostic or predictive value in HCC [21, 22].

In our study, VASN was screened as a differentially abundant component between HCC serum and normal control serum by subtractive EMSA-SELEX. It was further confirmed as a potential biomarker for HCC. Our results showed that the level of VASN was higher in sera of 100 cases of hepatocellular carcinoma compared with 97 normal cases and 129 cases of B type hepatitis, consistent with the higher expression of VASN in HCC cell lines and HCC tissues both at the mRNA and protein levels. No relationship between VASN and AFP was found, implying a combination of vasorin and AFP could increase the sensitivity of diagnosis. It is interesting that VASN, as a membrane protein, was found to promote cell proliferation and migration, which may also be a potential drug target for treatment of liver cancer.

## MATERIALS AND METHODS

### Reagents and antibodies

Taq polymeraseand dNTPs were purchased from TIANGEN BIOTECH Co Ltd, China. Quantitative vasorin ELISA kits were purchased from Uscn Life Science Inc, USA. Total RNA separation reagent-Trizol Kit was bought from Invitrogen company Co. Ltd, USA. DEPC, M-MLV reverse transcriptase were purchased from Promega Corporation, USA. SYBRGREEN PCR, Master Mix, quantitative PCR reagents were bought from Fermentas Corporation, USA. Mouse monoclonal antibody for VASN was prepared by Beijing C&N international SCI-Tech Co. Ltd, China and rat polyclonal antibody for VASN was purchased from Abnov company, USA. Mouse monoclonal GAPDH antibody and FITC labelled sheep antibody against rat IgG were productions of Santa Cruz Biotechnology, Inc., USA. The ECL kit and the QuantaRed^TM^ Enhanced Chemifluorescent HRP Substrate were bought from Thermo Fisher Scientific Inc, USA. Tissue qPCR array was bought from Ori Gene Biological Science and Technology Co. Ltd, USA.

### Random ssDNA library and primers

Random ssDNA library and primers were synthesized by Invitrogen Co Ltd, Beijing, China. The sequences are listed in Supplementary results Table 2. Primers Plong-1 and P11 were used for the standard PCR amplication of double-stranded DNA molecules. Primers Plong-1 and Pstem-loop were used to synthesize single-stranded DNA as in our previous published reference [16].

### Clinical samples and cell lines

Human liver cancer tissue, hepatitis tissue and hepatocirrhosis tissue samples were obtained from patients admitted for liver surgery who had serological tests for AFP or virus antigens/antibodies and pathologically confirmed diagnosis at the General Hospital of Fuzhou, China. The excised tissue samples were fixed in formalin and serially sectioned (4 μm). Human HCC serum samples and normal serum samples were collected from patients with confirmed diagnosis given at the Beijing YouAn Hospital, Institute of Hepatology Peking University and Tianjing Blood Center respectively. All the HCC diagnoses were verified by tumor biopsies or surgical pathology. The hepatitis B serum samples were collected at Peking University People's Hospital, Peking University Hepatology Institute and had confirmed diagnoses based on the Hepatitis B antigen/antibody test or nucleic acid tests. All of the serum specimens were obtained after consent was given by patients or tested normal persons. Human hepatoma cell line HepG2, SMMC-7721, and normal liver cell line HL-7702 (L02) were purchased from Shanghai Cell Bank of the Chinese Academy of Sciences. Human bladder cancer cell line T-24 was purchased from Beijing Cell Bank of Type Culture Collection of the Chinese Academy of Sciences. Human Laryngeal epidermoid carcinoma cell line Hep-2, lung cancer cell line A549 and breast cancer cell line MDA-MB-231 were from our laboratory store. All cells were cultured in Dulbecco's modification of Eagle's medium or Roswell Park Memorial Institute (RPMI) medium 1640 supplemented with 10% or 20% fetal bovine serum (Gibco, New York, USA).

### The subtractive – EMSA – SELEX

For the first round of selection, 1500 pmole of initial library was incubated with 5μl normal serum in binding buffer (PBS, 5mmol/L MgCl_2_) for 1 h. The complex was then separated by electrophoresis on an 8% native polyacrylamide gel. The nucleotides that did not bind to serum components and thus shifted equally with the same as the free library control were recovered from the gel. The recovered single stranded DNA was amplified through unequal length PCR^16^ to achieve a subtractive library. The subtractive library (400 pmole) was then subjected to positive selection: the pool was incubated with 5 μl target serum and the bound nucleotide which showed a slower mobility rate on the native PAGE gel was excised and recovered. Finally, recovered DNA was amplified by unequal length PCR to generate an enriched pool for the next round of selection. In this study, we performed the subtractive selection against one case of normal serum for the first, second and third rounds and against another case of normal serum on the fourth and fifth rounds. We executed positive selection to 1 case of AFP negative HCC serum through all five rounds. Supplementary Table 3 summarizes the detailed conditions for each round of selection. Selection rounds were repeated until there was an obvious binding of enriched pool with targets and the enriched pool could discriminate targets from counter-targets.

### Identification of the enriched selection pool

After each round of selection, the enriched pool was labeled with FAM using FAM modified primer Plong-1 through unequal length PCR. Enrichment of each round of selection was monitored by EMSA of labeled pool and targets or counter target complex, and visualized under laser excitation.

### Identification of candidate serological biomarkers

The enriched fifth pool and target serum were incubated and the complex were separated on 8% native polyacrylamide gel. The retarded band was excised and the protein components in it were recovered, digested with porcine trypsin and subjected to MALDI-TOF MS followed by secondary peptide sequencing MS analysis and a Mascot database search for the identification of the mixture proteins. The corresponding position in the control lane (the complex of the initial library and target serum) was excised and identified by MS simultaneously to exclude the protein mobilized similarly with the complex. The candidate serological biomarkers of hepatoma were thus gained.

### Detection of expression of VASN in serum of liver cancer by ELISA

The VASN level in serum samples from 100 patients with liver cancer and 97 healthy persons obtained clinically were detected with the quantitative ELISA kit according to manufacturer's instructions except that we used the QuantaRed^TM^ Enhanced Chemifluorescent HRP Substrate for higher sensitivity. Briefly, 100 μl substrate solution was added to each well, incubated for no longer than 20 min at 37°C and protected from light. 10 μl stop solution was added when gradient color changes were obvious in the first 3-4 wells while not in the last 3-4 wells. Finally, the reaction was read spectrophotmetrically at ~570nm.

### Silencing VASN expression

siRNAs to VASN and negative control, miRNA mimics and inhibitors were synthesized by Guangzhou ruibo company. The transfection of small RNAs was conducted in 6-well plates using the Lipofectamine 2000 transfection reagent (Invitrogen, Carlsbad, CA) following the manufacturer's manual. Inhibitors to miRNA were transfected at a molecular ratio of 3:1 to miRNA. The total RNA and the whole cell lysate was prepared 48 h after transfection and subjected to real-time PCR or Western Blot.

### Overexpressing VASN in normal hepatocyte

The cDNA of VASN (GenBank accession number NM­_138440) was obtained by reverse transcription from HepG2 total RNA using the M-MLV Reverse Transcriptase Kit according to manufacturer's manual. The cDNA was amplified and subsequently inserted into vector pEGFP N1. Clones obtaining the expression plasmid pEGFP N1/VASN were confirmed by sequence analysis. The plasmid was transfected into normal hepatocyte L02 cell lines using lipofection reagent to gain a transient overexpression of vasorin. The expression was confirmed by fluorescent microscope imaging and Western Blot 48 h after transfection.

### Immunofluorescence assay

Tissue slides and coverslips with adherent cells were pre-incubated with blocking reagents (2% BSA) and then incubated with VASN antibody (1:100 dilution) at 37°C for 2 h, followed by incubation with FITC-labeled goat anti-mouse secondary antibody (1:50 dilution) at 37°C for 1 h. 0.25% Evans blue dye was used to eliminate nonspecific fluorescent background. 100 ng/ml DAPI was used to stain the nucleus. The fluorescence patterns of each experimental group were scanned with a Zeiss LSM 510META confocal microscope or fluorescence miscroscope using the same instrument settings.

### Detection of expression of VASN in hepatocarcinoma tissues by Q-PCR

Tissue cDNA chips including cDNA from 68 cases of liver cancer tissue, pericarcinoma tissues and hepatocirrhosis that were β-actin calibrated were purchased. The expression of VASN mRNA was measured by real-time PCR.

### Prediction of miRNAs targeting VASN

Online bioinformatic software (http://www.targetscan.org) was used to predict of the potential miRNAs regulating VASN and potential miRNA target sites in the 3′ UTR of VASN gene.

### Real-time RT-PCR

Total RNA was extracted from cells or serum samples using the TRIzol LS Reagent (Invitrogen, Carlsbad, CA, USA) according to the manufacturer's instructions. The concentration of total RNA was measured by a GeneQuant spectrophotometer (GE Healthcare Life Sciences, USA). cDNA was generated using the M-MLV Reverse Transcriptase Kit (Promega, USA) according to the manufacturer's instructions. Real-time RT-PCR was performed with SYBR Green PCR Kit (Promega, USA) on an Mx3000P QPCR System (Stratagene, La Jolla, CA, USA). CT values were determined using Mx3000p software (version 4.10) with an amplification-based threshold determination and adaptive baseline analysis options.

### Assay of luciferase activity

The VASN 3′ UTR was amplified by PCR from genomic DNA of HepG2 cells and cloned into the pGL3-control vector (Promega, USA) to construct vasorin 3′ UTR miRNA reporter plasmids. Mutant sequences were produced by a Mutagenesis Kit (Takara, USA) and inserted into the pGL3-control vector to construct vasorin 3′ UTR miRNA mutant reporter plasmids. HepG2 cells were seeded in 24-well plates and transfected in triplicate with luciferase reporters (1 μg), pRL-CMV (10 ng, as a control for transfection efficiency and sample handling) and miRNA mimics (100 pmol). Cells were harvested at 48 h post-transfection and assayed with Dual Luciferase Assay (Promega, USA) according to the manufacturer's instructions.

### Western blot

Preparation of whole-cell protein lysates and Western blot analysis were performed as described previously. Briefly, 80 μg of total protein was loaded and separated on sodium dodecyl sulfate-polyacrylamide gel electrophoresis (SDS-PAGE), using 10% acrylamide gels, then transferred to polyvinylidine diﬂuoride (PVDF). The expression of VASN was determined using primary anti-VASN antibody (1:500 dilution) followed by HRP-goat anti-mouse/rabbit IgG. The blots were detected using the enhanced chemiluminescence Western blotting detection system.

### MTS cell proliferation assay

Cells were seeded in 96-well plate at a density of 2×10^3^ per well in 100 μl media and cultured for 24 h to reach a 40–50% confluency. The cells were transfected with small RNA molecules or DNA constructs and cultured for a further 24-96 h. 20 μl MTS(CellTiter 96AQ_ueous_ Non-Radioactive Cell Proliferation Assay, Promega, USA) and 100 μl fresh medium were added into each well and then incubated for 1 h. The absorbance of the wells was determined using a plate reader at a test wavelength of 490 nm.

### Scratch healing assay

Cells were seeded in a 6-well plate at a density of 5×10^5^ per well in 2 ml media and cultured overnight. At 60–70% confluency, the cells were transfected with small RNA molecules or DNA constructs and cultured for a further 24 h. Upon reaching 90% confluency, a scratch was created with a 200 μl-pipette tip and wells were washed three times with PBS to remove loose cells. Then cells were incubated in fresh medium and cell scratches were photographed every 6 h.

### Migration assay

Cells were seeded in 6-well plates, transfected with nucleotide acids of interest and cultured for further 24 h as described before. The cells were then cultured in serum-free medium for another 24 h. Cells were detached with trypsin, harvested by centrifugation, resuspended in complete medium and seeded in the upper chamber of a Transwell (Corning, NY, USA) at a density of 5×10^4^ in 100 μl media, and placed in a 24-well plate containing 500 μl complete medium. After incubation for 12-24 h at 37°C, the cells on the bottom side of the chamber were ﬁxed with formaldehyde for 30 min, then stained by crystal violet or DAPI as described. Migrating cells were counted manually in four random microscopic fields.

### Apoptosis assay

Cells of different treatment were harvested by trypsinization and washed with ice-cold PBS twice. Apoptotic cells were stained using the Annexin V FITC apoptosis detection kit (MULTISCIENCES, China) and PI according to manufacturer's instructions and detected by flow cytometry (BD FACSCalibur, USA).

## SUPPLEMENTARY MATERIALS, TABLES


