# Supplementary material for: Vasorin is a potential serum biomarker and drug target of hepatocarcinoma screened by subtractive-EMSA-SELEX to clinic patient serum
Source: Oncotarget. 2015 Mar 12;6(12):10045–59. doi: 10.18632/oncotarget.3541 (PMC4496339; doi:10.18632/oncotarget.3541)
Supplement: Supplementary file 1 [file oncotarget-06-10045-s001.pdf]

Vasorin is a potential serum biomarker and drug target of hepatocarcinoma screened by subtractive-EMSA-SELEX to clinic patient serum

Supplementary Material

Table 1: Potential biomarkers identified by MS

GI	Protein	Matched Peptides	Peptide	Proteins (GI) matching the same set of peptides
gi|1620909	Ceruloplasmin	5	K.DIASGLIGPLIICK.K+ (C)<br>K.VNKDDEEFIESNK.M<br>K.DLYSGLIGPLIVCR.R+ (C)<br>K.LISVDTEHSNIYLQNGPDR.I<br>R.GPEEEHLGILGPVIWAEVGDTIR.V	ceruloplasmin precursor or isoforms: (gi|4557485) (gi|119599289) (gi|119599290) (gi|126031006) (gi|158255874) (gi|221042622)
gi|291922	complement factor B	4	K.VASYGVKPR.Y<br>K.VSEADSSNADWVTK.Q<br>R.DFHINLFQVLPWLK.E<br>K.EAGIPEFYDYDVALIK.L	Complement factor B preproprotein or Segment (gi|13278732) (gi|58176651) (gi|67782358) (gi|134105218) (gi|194384366) (gi|239781743) (gi|251837060)
gi|194384410	unnamed protein product	3	K.EYVLPSFEVIVEPTEK.F<br>K.VQLSNDFDEYIMAIEQTIK.S<br>R.EPGQDLVVLPLSITTDFIPSFR.L	complement component C3 or precursor (gi|179665) (gi|115298678)
gi|15489339	VASN protein	1	R.LLLLDLSHNSLLALEPGILDTANVEALR.L	CSRV314 or slit-like 2 precursor (gi|37181716) (gi|37181718) (gi|88702793)
gi|106529	Ig kappa chain C region	1	K.SGTASVVCLLNNFYPR.E+ (C)	Immunoglobulin (gi|125145) (gi|184848) (gi|185925) (gi|185927) (gi|185947) (gi|185949) (gi|229526) etc
gi|386789	hemopexin precursor	3	R.RLWWLDLK.S<br>R.YYCFQGNQFLR.F+ (C)<br>K.EVGTPHGIILDSVDAAFICPGSSR.L+ (C)	hemopexin precursor or unnamed protein (gi|1335098) (gi|11321561) (gi|189053897)
gi|51173528	carboxypeptidase N precursor	1	K.TLNLAQNLLAQLPEELFHPLTSLQTLK.L	Carboxypeptidase N, polypeptide 2 (gi|52788240) (gi|119598461) (gi|145207281) (gi|256217721)

Supplementary results Table 2: The VASN level in 326 tested individuals for the ELISA analysis

HCC			Normal		Hepatitis	
Case No	VASN (ng/ml)	AFP （TU）	Case No	VASN (ng/ml)	Case No	VASN (ng/ml)
1	14.70647	444.3	1	2.483388	1	9.044036
2	12.45442	1000	2	2.307037	2	4.607394
3	10.57283	830.8	3	2.203464	3	4.458231
4	8.350219	1000	4	2.169312	4	4.324461
5	6.863982	1000	5	2.135347	5	3.72238
6	6.448114	1000	6	2.085306	6	3.579855
7	6.345765	494.8	7	2.040221	7	3.47553
8	5.983209	4.6	8	2.03457	8	3.325962
9	5.967843	1.25	9	1.968317	9	3.147103
10	5.796683	1000	10	1.939469	10	2.930384
11	5.302034	34.21	11	1.870335	11	2.866855
12	4.994912	3.41	12	1.495186	12	2.866855
13	4.994912	3.41	13	1.465135	13	2.383298
14	4.994912	1000	14	1.385336	14	2.383298
15	4.76476	3.07	15	1.376101	15	2.341935
16	4.506769	2.51	16	1.376101	16	2.231864
17	4.385359	168	17	1.346795	17	2.203464
18	4.264199	0.819	18	1.33496	18	2.157976
19	4.264199	1000	19	1.309772	19	2.101568
20	4.22191	276.7	20	1.294024	20	2.067976
21	3.86626	1000	21	1.234208	21	1.908964
22	3.84211	1000	22	1.203065	22	1.891749
23	3.782062	1.52	23	1.174878	23	1.87461
24	3.722076	1.36	24	1.146878	24	1.82365
25	3.722076	1000	25	1.119064	25	1.805946
26	3.542495	1.7	26	1.114793	26	1.691109
27	3.482759	1000	27	0.98279	27	1.691109
28	3.423087	1.7	28	0.98279	28	1.642663
29	3.363476	4.22	29	0.957142	29	1.626667
30	3.155944	1000	30	0.929585	30	1.610747
31	3.108958	1000	31	0.906766	31	1.594903
32	3.068803	996.3	32	0.903262	32	1.579135
33	2.947	34.77	33	0.887882	33	1.532289
34	2.888846	0.766	34	0.881578	34	1.465135
35	2.864196	839.3	35	0.876	35	1.425648
36	2.787898	582.7	36	0.864193	36	1.381088
37	2.742125	1.49	37	0.851175	37	1.351762

38	2.602019	1000	38	0.829232	38	1.32274
39	2.418728	5.16	39	0.825412	39	1.32274
40	2.3497	1000	40	0.806014	40	1.25999796
41	2.210712	18.28	41	0.780826	41	1.25152
42	2.210712	1000	42	0.774444	42	1.155011
43	2.184408	2.58	43	0.755639	43	1.141529
44	2.174863	802.8	44	0.730451	44	1.128123
45	2.174853	1000	45	0.728461	45	1.114793
46	2.126112	2.07	46	0.724221	46	1.091436
47	2.126112	13.57	47	0.696242	47	1.07526
48	2.067878	41.95	48	0.685654	48	1.063995
49	2.058681	3.02	49	0.680075	49	1.062235
50	2.009706	0.629	50	0.680075	50	1.036740014
51	2.009706	1.14	51	0.674744	51	1.036413
52	1.998282	1000	52	0.664708	52	1.023617
53	1.981598	5.02	53	0.664708	53	1.009672
54	1.951598	95.05	54	0.644067	54	0.98278954
55	1.94505	178	55	0.63386	55	0.960776
56	1.897027	1000	56	0.63386	56	0.936174
57	1.86128	16.37	57	0.626013	57	0.903261837
58	1.812878	1.27	58	0.613676	58	0.899841
59	1.7946	1000	59	0.613676	59	0.899841
60	1.77942	1000	60	0.603698	60	0.887882
61	1.777646	802.8	61	0.603698	61	0.876
62	1.763478	1000	62	0.601927	62	0.864193
63	1.719007	1000	63	0.583971	63	0.852463
64	1.661992	1000	64	0.579323	64	0.852463
65	1.608508	2.01	65	0.578028	65	0.829232
66	1.604258	10.55	66	0.574222	66	0.829232
67	1.58483	420.6	67	0.564548	67	0.81773
68	1.550826	881.6	68	0.554314	68	0.806305
69	1.51708	1000	69	0.530787	69	0.794956
70	1.394315	2.87	70	0.530787	70	0.794956
71	1.357694	1000	71	0.526618	71	0.783683
72	1.285037	49.5	72	0.508109	72	0.783683
73	1.259171	1.52	73	0.507447	73	0.774444
74	1.259171	1000	74	0.507447	74	0.772486
75	1.201875	1000	75	0.498969	75	0.772486
76	1.087472	486.1	76	0.489906	76	0.761365
77	1.00646	32.08	77	0.472007	77	0.750321
78	0.965261	22.04	78	0.472007	78	0.750321
79	0.954128	1000	79	0.472007	79	0.739353
80	0.856548	6.91	80	0.472007	80	0.728461
81	0.8418	508.6	81	0.472007	81	0.728461

82	0.804254	1.99	82	0.453384	82	0.728461
83	0.804254	4.22	83	0.438544	83	0.717645
84	0.76495	0.847	84	0.437123	84	0.717645
85	0.726025	154.3	85	0.420139	85	0.717645
86	0.687479	1.41	86	0.415949	86	0.706905
87	0.649313	1.44	87	0.411761	87	0.706905
88	0.63236	7.18	88	0.393541	88	0.706905
89	0.630519	1000	89	0.37901	89	0.706905
90	0.549244	1000	90	0.371013	90	0.654349
91	0.537087	0.662	91	0.352632	91	0.62373
92	0.464166	3.61	92	0.277069	92	0.593796
93	0.462726	2.23	93	0.241901	93	0.593796
94	0.441766	470.9	94	0.241901	94	0.583971
95	0.392761	1.9	95	0.241901	95	0.578027518
96	0.266145	23.87	96	0.220984	96	0.574222
97	0.254501	3.67	97	0.079783	97	0.574222
98	0.122306	5.45			98	0.564548
99	0.058483	2.62			99	0.554314301
100	0.013119	126.4			100	0.535986
					101	0.530787486
					102	0.526618
					103	0.507447
					104	0.484293
					105	0.484293
					106	0.480918
					107	0.463172
					108	0.463172
					109	0.438544
					110	0.411761
					111	0.411761
					112	0.403459
					113	0.395233
					114	0.395233
					115	0.395233
					116	0.393541
					117	0.37901
					118	0.371319
					119	0.371013
					120	0.371013
					121	0.371013
					122	0.347479
					123	0.347479
					124	0.327434205
					125	0.309779

126	0.28099
127	0.240092
128	0.220984
129	0.159351

The vasorin level higher than cut-off value (1.5061ng/ml) was labeled in red；The AFP level under 5.8TU was considered to be negative.

Supplementary results Table 3: The immunofluorescence result of 7 cases liver tissues

	Cases					
Diseases	-	+	++	+++	++++	Total
HCC		1	0	2	1	4
hepatitis	1	1	0	0	0	2
hepatocirrhosis	1	0	0	0	0	1

-: <5% of cells stained; +: 5%-25% of cells stained; + +: 26%–50% of cells stained; + + +: 51%–75% of cells stained; + + + +: >75% of cells stained.

Supplementary results Table 4: Random ssDNA library and primers were used in the experiments.

Name	Sequence
GP35	5′-G CAATGGTACGGTACTTCC-N35-CAAAAGTGCACGCTACTTTGCTAA-3′
Plong-1	5′ - GCAATGGTACGGTACTTCC-3′
P11	5′- TTAGCAAAGTAGCGTGCACTTTTG-3′
Pstem-loop	5′- GCTAAGCGGGTGGGACTTCCTAGTCCCACCCGCTTAGCAAAGTAGCGTGCACTTTTG-3′
qPCR-Vasorin-F	5′- GCTCAAGCTGCAGGACAAC-3′

qPCR-Vasorin-R	5′- GAAGAGCCCCTCGTCCAG-3′
qPCR-β-actin-F	5′- GGCATCGTGATGGACTCCG-3′
qPCR-β-actin-R	5′- GCTGGAAGGTGGACAGCGA-3′
miR145-F	5′- GTCCAGTTTTCCCAGGAATCCCT-3′
miR145-R	5′- GCGAGCACAGAATTAATACGAC-3′
miR146a-F	5′- GCTGAGAACTGAATTCCATG -3′
miR146a-R	5′- GCGAGCACAGAATTAATACGAC-3′
miR146b-F	5′- CGGCGTGAGAACTGAATTCCAT-3′
miR146b-R	5′- GCGAGCACAGAATTAATACGAC-3′
miR205-F	5′- TCCTTCATTCCACCGGAGTCTG -3′
miR205-R	5′- GCGAGCACAGAATTAATACGAC -3′
miR214-F	5′- ACAGCAGGCACAGACAGGCAGT -3′
miR214 -R	5′- GCGAGCACAGAATTAATACGAC-3′
miR761-F	5′- CGGCAGCAGGGTGAAACTG -3′
miR761-R	5′- GCGAGCACAGAATTAATACGAC-3′
miR3619-F	5′- TCTCAGCAGGCAGGCTGGT -3′
miR3619-R	5′- GCGAGCACAGAATTAATACGAC -3′
FL-VASN-F	5′- GCTCAAGCTGCAGGACAAC-3′
FL-VASN-R	5′-GAAGAGCCCCTCGTCCAG -3′
VASN 3’UTR-F	5′- TGCTCTAGAGCCAGAGAGAGACAGG-3′
VASN 3’UTR-R	5′- CCGGAATTCAAACACACTTCATCTT-3′
VASN-145-1 mu F	5′- TGCTCTAGA GCCAGAGAGAGACAGG -3′

VASN-145 1 mu R	5′- TAAAGCATCTTCCTTTCTTTTGTTCCTTCGC-3′
VASN-145-2 mu F	5′- CGAA GGAACAAAAG A AAGGAAGATGCTTTAG -3′
VASN-145-2 mu R	5′- CCGGAATTC AAACACACTTCATCTT -3′
VASN-146a mu F	5′- TGCTCTAGAAGTCCCAACCTCGGGG-3′
VASN-146a mu R	5′- CCGGAATTCAAACACACTTCATCTT -3′

Supplementary results Table 5: The detailed conditions for each round of subtractive EMSA-SELEX selection.

Round	ssDNA pool (pmol)	AFP negative serum (μl)	Normal serum (μl)	PCR cycle	Subtractive Incubated time (h)	Incubated time (h)
1	1500	5	5	35	1	1
2	300	5	5	35	1	1
3	120	5	5	35	1	1
4	120	5	5	30	1	1
5	120	5	5	30	1	1
